# p*K*_a_ prediction in non‐aqueous solvents

**DOI:** 10.1002/jcc.27517

**Published:** 2024-12-11

**Authors:** Jonathan W. Zheng, Emad Al Ibrahim, Ivari Kaljurand, Ivo Leito, William H. Green

**Affiliations:** ^1^ Department of Chemical Engineering Massachusetts Institute of Technology Cambridge Massachusetts USA; ^2^ Institute of Chemistry University of Tartu Tartu Estonia

**Keywords:** acid dissociation, COSMO‐RS, ions, pKa, solvation

## Abstract

Acid dissociation constants ($pK_{a}$) are widely measured and studied, most typically in water. Comparatively few datasets and models for non‐aqueous $pK_{a}$ values exist. In this work, we demonstrate how the $pK_{a}$ in one solvent can be accurately determined using reference data in another solvent, corrected by solvation energy calculations from the COSMO‐RS method. We benchmark this approach in 10 different solvents, and find that $pK_{a}$ values calculated in six solvents deviate from experimental data on average by less than 1 $pK_{a}$ unit. We observe comparable performance on a more diverse test set including amino acids and drug molecules, with higher error for large molecules. The model performance in four other solvents is worse, with one MAE exceeding 3 $pK_{a}$ units; we discuss how such errors arise due to both model error and inconsistency in obtaining experimental data. Finally, we demonstrate how this technique can be used to estimate the proton transfer energy between different solvents, and use this to report a value of the proton's solvation energy in formamide, a quantity that does not have a consensus value in literature.

## Graphical abstract

The acid dissociation constant (p*K*_a_) plays a crucial role in applications ranging from pharmaceutical synthesis to environmental science. This work demonstrates that popular solvation model COSMO‐RS can be used in conjunction with aqueous p*K*_a_ data to predict the p*K*_a_ in many non‐aqueous solvents, which can then be leveraged to approximate the transfer free energy of the proton between solvents. This work also explores inconsistencies in experimental data that sometimes lead to worse model agreement.

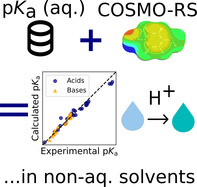

## 1 INTRODUCTION

The acid dissociation constant, or $pK_{a}$, is implicated in the behavior of pharmaceutical drugs in the human body, environmental impact of molecules, and other applications of chemistry.^1^, ^2^, ^3^ In recent years, several models^4^, ^5^, ^6^, ^7^, ^8^, ^9^ and open‐source data compilations^10^, ^11^, ^12^, ^13^, ^14^, ^15^ have been developed for aqueous $pK_{a}$ predictions. Such developments in non‐aqueous solvents are comparatively fewer.^16^, ^17^

In light of the fewer available data, a variety of approaches have been proposed to calculate non‐aqueous $pK_{a}$ values. One approach is to train a neural network in a single solvent by augmenting the training set with computed values.^18^ Another approach involves training a deep learning model simultaneously across multiple solvents. This method was used on the iBonD dataset, which includes more than 30,000 $pK_{a}$ data distributed across 46 solvents.^17^ This approach was reported to score an overall MAE of 0.89 $pK_{a}$ units, though the errors associated with specific solvents were higher, with MAEs in DMSO and acetonitrile respectively exceeding 1.5 and 1.2 $pK_{a}$ units.^19^ The full data corpus, although accessible through a website and much larger than all other non‐aqueous $pK_{a}$ compilations, is not available in a convenient format for data science applications. Therefore, the potential of further extending machine learning approaches is also limited by the low availability of open‐source experimental data.

One other class of models is to relate the $pK_{a}$ in the desired solvent to a calculable or measurable energy difference. In this way, the $pK_{a}$ is accessed via a thermodynamic cycle. A variety of relations have been proposed, relating $pK_{a}$ to computed Gibbs free energies of dissociation,^18^, ^20^, ^21^ computed $pK_{a}$ values,^22^ and experimental $pK_{a}$ values in the same solvent^23^ or a reference solvent (typically water).^24^, ^25^, ^26^ In all of these approaches, a linear model is constructed wherein empirical linear parameters are calculated. A varying degree of error is introduced in the choice of regression variable. Using the $pK_{a}$ of the analyte in water as the regression variable has the benefit of low data uncertainty, as experimental aqueous $pK_{a}$ values are often reported to less than 0.2 $pK_{a}$ units.^12^, ^13^, ^14^, ^15^ Therefore, we chose to use the reference solvent method in this work with water as the reference solvent.

**FIGURE 1:**
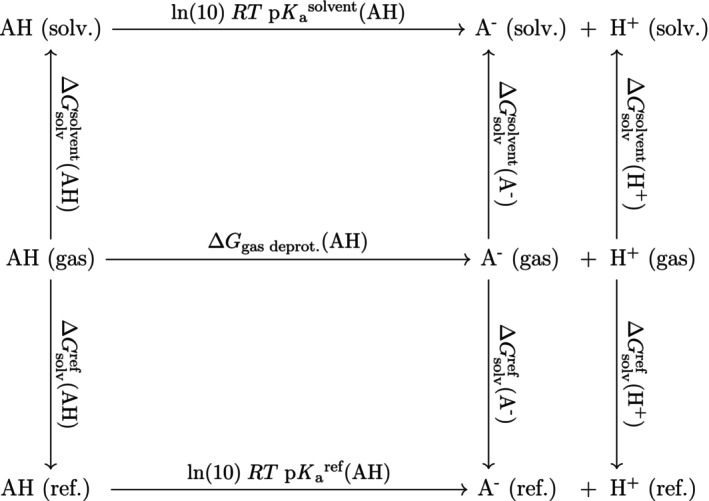
Thermodynamic cycle for the dissociation of a neutral acid in different phases.

**FIGURE 2:**
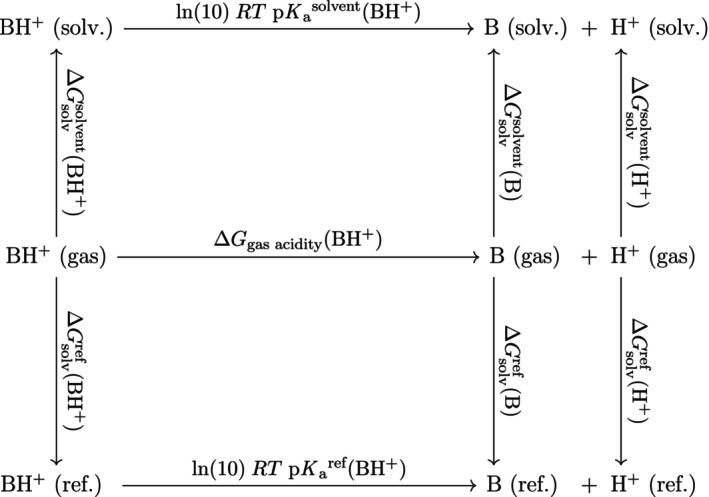
Thermodynamic cycle for the dissociation of a cation acid in different phases.

It is sometimes useful to utilize the thermodynamic cycle of gas‐phase dissociation followed by solvation, shown in Figures 1 and 2. This formulation links the $pK_{a}$ to the solvation energy of the proton, which has only been measured in a handful of solvents^26^, ^27^ but is required to establish the pH scale in different solvents. For non‐aqueous $pK_{a}$ data, such proton solvation free energies are either implicitly assumed, or explicitly assigned. For many solvents, this term can be recovered from the $pK_{a}$ data if treated as a regression parameter. For example, Rossini et al.^28^, ^29^ estimated the proton's solvation energy in water, acetonitrile, methanol, and acetone by using a linear regression comparing computed and experimental $pK_{a}$ values. Then, they used those proton solvation energies with the reference solvent method to compute $pK_{a}$ values with RMSDs within 0.8 $pK_{a}$ units.^25^ In their works, the authors used the electrostatic solvation model SOLVATE within the MEAD software suite.^31^, ^32^

In this work, we benchmark a procedure for calculating $pK_{a}$ values in 10 different solvents by reference to data in water. We use the conductor‐like screening model COSMO‐RS, which has previously been demonstrated to successfully compute $pK_{a}$ values in a handful of solvents such as acetonitrile^20^ and acetone.^21^ We show that this approach only requires one regression parameter, which can be used to approximate the proton's solvation energy in each new solvent. One considerable obstacle in predicting non‐aqueous $pK_{a}$ values is the issue of experimental error. $pK_{a}$ values are often measured relative to each other and then “anchored” to a reference value, due to the ambiguity surrounding the solvation free energy of the proton, as well as experimental considerations especially in weakly‐screening solvents. An important consideration is solvent purity, in particular water content, which can significantly affect $pK_{a}$ measurements especially in low‐polarity aprotic solvents. The choice of reference value can lead to significant inconsistencies among data sources; discrepancies of 3 $pK_{a}$ units, and sometimes more, are not uncommon.^21^ Experimental error can also arise if ion pairing (wherein ions form pairs or clusters, especially in weakly‐screening solvents) and homoconjugation (wherein anions may form adducts with neutral acids) are not accounted for.^33^ There is also systematic error in solvation free energy computations for ionic compounds, which are present in this workflow.^34^ Hence, model error must inextricably be considered alongside experimental error, and the relative contribution of each is not always clear. In this work, we attempt to correct for these issues by curating experimental data. We discuss a few examples wherein remaining inconsistencies in experimental data appear to be responsible for significant deviations from the model predictions, as well as several cases in which the situation is less clear.

## 2 METHODS

### 2.1 Dataset selection

We utilized $pK_{a}$ data presented by Busch and collaborators, which includes a compilation of $pK_{a}$ data from the iBonD dataset.^22^ From their data spanning 18 solvents, we chose solvent systems with at least 10 data points and with representation of both acids and bases, resulting in a set of 10 solvents. We further modified the dataset by removing doubly‐charged species, and selected only species with both aqueous and non‐aqueous $pK_{a}$ values. The selected compounds are all small molecules, consisting nearly entirely of substituted benzoic and phenolic acids, alkyl carboxylic acids, alkylamines, and pyridine and aniline derivatives.

We then critically curated the data to ensure consistency and accuracy of the data in all solvents. In acetonitrile, DMSO, DMF, pyridine, and acetone, we replaced some values from the Busch collection with trusted values from the literature. More information about the data curation can be found in the Supporting Information.

Table 1 shows the number of data available per solvent. All solvents include at least 10 data points. Some solvent systems (acetonitrile, DMSO, and methanol) include more than 45 datapoints and are split roughly half‐half between acids and bases.

**TABLE 1:** Datapoints per solvent.

Solvent	# of acids	# of bases
Acetone	15	4
Acetonitrile	20	27
DCE	12	3
DMF	17	5
DMSO	30	24
Ethanol	19	14
Formamide	9	12
Methanol	24	27
Nitromethane	5	17
Pyridine	12	0

### 2.2 Calculating $pK_{a}$ in different solvents

The $pK_{a}$ in one solvent is calculated by applying solvent corrections to $pK_{a}$ data in a reference solvent (water). For acids, this relationship is:

(1) $$p{K_{a}}^{\text{solvent}}=p{K_{a}}^{\mathrm{ref}}+\frac{1}{\ln\left(10\right)\mathrm{RT}}\left(\Delta \Delta G_{\text{solv}}^{†}\left(H^{+}\right)+\Delta \Delta G_{\text{solv}}^{†}\left(A^{-}\right)-\Delta \Delta G_{\text{solv}}^{†}\left(\mathrm{AH}\right)\right),$$

and for bases,

(2) $$p{K_{a}}^{\text{solvent}}=p{K_{a}}^{\mathrm{ref}}+\frac{1}{\ln\left(10\right)\mathrm{RT}}\left(\Delta \Delta G_{\text{solv}}^{†}\left(H^{+}\right)-\Delta \Delta G_{\text{solv}}^{†}\left({\mathrm{BH}}^{+}\right)+\Delta \Delta G_{\text{solv}}^{†}\left(B\right)\right),$$

where $\Delta \Delta G_{\text{solv}}^{†}\left(Z\right)\equiv \Delta G_{\text{solv}}^{\text{solvent}}\left(Z\right)-\Delta G_{\text{solv}}^{\mathrm{ref}}\left(Z\right)$. In these equations, RT is the product of the molar gas constant with temperature. The temperature is assumed to be 298 K. $\Delta G_{\text{solv}}\left(Z\right)$ is the solvation energy of a solute at an arbitrary reference state. $A^{-}$ refers to an anionic base and AH refers to its protonated conjugate acid. Likewise, ${\mathrm{BH}}^{+}$ refers to a cationic acid and B refers to its deprotonated conjugate base. $pK_{a}^{\text{solvent}}$ refers to the dissociation constant of a molecule in a non‐aqueous solvent of interest (i.e., the solvents in Table 3), and $pK_{a}^{\mathrm{ref}}$ refers to the dissociation of that same species in water. In this work, the † superscript denotes a reference value of water.

Because $\Delta \Delta G_{\text{solv}}^{†}\left(H^{+}\right)$ is not known, we treated the term as a regression parameter, $\delta_{H}^{†}$, that minimizes the absolute residual of the fit for each solvent:

(3) $$f_{i}\left(\delta \right)=\{\begin{array}{cc} pK_{a_{i}}^{\mathrm{ref}}-pK_{a_{i}}^{\text{solvent}}+\frac{1}{\ln\left(10\right)\mathrm{RT}}\left(\delta -∆∆G_{\text{solv}}\left({\mathrm{BH}}_{i}^{+}\right)+∆∆G_{\text{solv}}\left(B_{i}\right)\right) & \text{p}{\text{K}}_{a_{i}}=\text{basic}\\ pK_{a_{i}}^{\mathrm{ref}}-pK_{a_{i}}^{\text{solvent}}+\frac{1}{\ln\left(10\right)\mathrm{RT}}\left(\delta -∆∆G_{\text{solv}}\left(A_{j}^{-}\right)+∆∆G_{\text{solv}}\left({\mathrm{AH}}_{i}\right)\right) & \text{p}{\text{K}}_{a_{i}}=\text{acidic} \end{array},$$

(4) $$\delta_{H}^{†}={\text{argmin}}_{\delta }\left(\sqrt{\sum_{i}^{N}f_{i}{\left(\delta \right)}^{2}}\right),$$

where i refers to the index of the neutral form of the acid or base under consideration, N is the number of datapoints per solvent, δ is a proxy estimate for the proton transfer energy, and $f_{i}\left(\delta \right)$ is the loss function that corresponds to the optimal $\delta_{H}^{†}$ when minimized. In Equation (3), the $pK_{a}$ terms are experimental values, whereas the $\Delta \Delta G_{\text{solv}}$ terms are computed using COSMO‐RS.

One advantage to this method is that it does not require any assumption of the absolute reference solvation energy $\Delta G_{\text{solv}}^{\mathrm{ref}}\left(H^{+}\right)$, which is prone to significant error. Additionally, the solvation terms are likely to include cancellation of model error, as one $pK_{a}$ calculation involves energy differences between different solvents (in each $\Delta \Delta G_{\text{solv}}$ term) and also within the same solvent (by taking the difference of solvation energies between the acid and its conjugate base).

Each $\Delta \Delta G_{\text{solv}}$ term describes the partitioning of the solutes between different phases—this energy difference is also termed the *transfer free energy*, and is directly proportional to log(*P*). The COSMO‐RS method has been previously benchmarked against the SAMPL challenges for log(*P*) of small neutral drug‐like molecules, with root mean squared deviations of around 0.7 $pK_{a}$ units overall, and with model performance depending on the solvent.^36^, ^37^ One such study observed higher errors for log(*D*), which accounts for ionization.^38^ The SM8 implicit solvation model has been evaluated on log(*P*) for organic solutes between water and other solvents, with mean unsigned errors reported to exceed 3 log units.^39^, ^40^ Given the high uncertainty in computing transfer free energies, particularly for ionic solutes, the accuracy of this method is dependent on the degree of error cancellation.

If $pK_{a}$ data for a compound is available in multiple solvents, it is possible to compute the $pK_{a}$ using an ensemble of reference values rather than using a single reference. The fitting procedure can also be done simultaneously with multiple references utilizing all available data for a given solvent (see Supporting Information).

### 2.3 QM details

A large set of initial conformers of each molecule was constructed using CREST^41^ with GFN2‐xTB version 6.6.0^42^ using the analytical linearized Poisson‐Boltzmann (ALPB) model^43^ with water as the solvent. The 20 lowest‐energy conformers for each species were re‐optimized using TURBOMOLE v7.7^44^ at the BP86^45^, ^46^/def2‐TZVP^47^ level of theory with the COSMO model at the conductor limit (ϵ=∞). These 20 conformers were further filtered by pruning conformer geometries based on dihedral angles, removing conformers that matched dihedral angles with a maximum absolute deviation less than 10 degrees and a root mean square deviation within 20 degrees. The conformers were also screened using the Python package RDMC^48^ to ensure that their 3D geometries correspond to their molecular graphs. The σ‐profiles and single‐point gas‐phase energies of the remaining optimized conformers were then calculated using TURBOMOLE at the BP‐TZVPD‐FINE level. COSMO‐RS was then used to calculate the solvation energies at 298 K via COSMOtherm 2023.^49^, ^50^

Optimized geometries were then manually inspected to ensure that they converged to reasonable structures. Geometries for all species were constructed independently using this approach (i.e., optimized conformers for neutral species were not used to generate initial geometries for ionic conjugate acids/bases). The optimized geometries are included in the SI.

## 3 RESULTS

### 3.1 Evaluation of model on parameterization data

**FIGURE 3:**
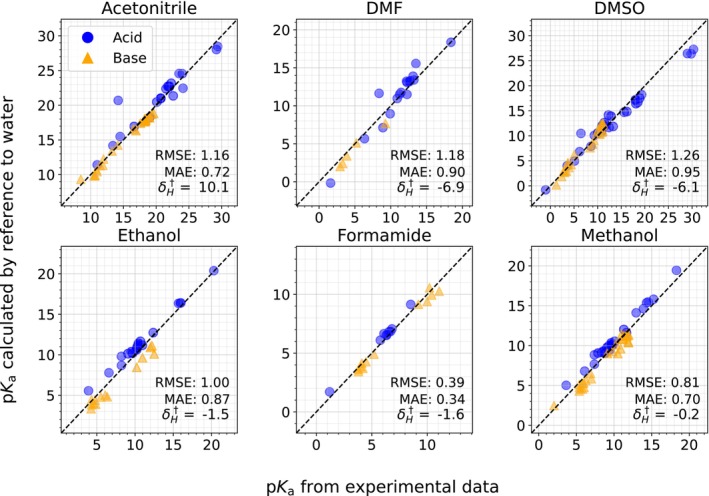
$pK_{a}$ values calculated relative to a reference $pK_{a}$, for the six solvents with MAEs less than 1 $pK_{a}$ unit. Blue circles refer to acidic $pK_{a}$ (involving anions) and orange triangles to basic $pK_{a}$ (involving cations). The RMSE and MAE are shown in $pK_{a}$ units, and the corresponding $\delta_{H}$ in kcal mol^−1^.

The $pK_{a}$ of acids and bases in six solvents (acetonitrile, DMF, DMSO, ethanol, formamide, and methanol) were calculated to MAEs within 1 $pK_{a}$ unit (Figure 3). Model errors were higher in acetone, nitromethane, pyridine, and dichloroethane. A summary of the error statistics is shown in Table 2. The six best‐performing solvents all include *R* ^2^ values exceeding 0.9, MAEs less than 1 $pK_{a}$ unit, and RMSEs less than 1.3 $pK_{a}$ units. The best performance was seen in formamide, with an RMSE of 0.39 $pK_{a}$ units.

**TABLE 2:** Error Statistics of $\delta_{H}^{†}$ and $pK_{a}$.

Solvent	$\delta_{H}^{†}$ (kcal mol^−1^)	RMSE	MAE	*R* ^2^
Acetonitrile	10.1	1.16	0.72	0.94
DMF	−6.9	1.18	0.90	0.92
DMSO	−6.1	1.26	0.95	0.96
Ethanol	−1.5	1.00	0.87	0.93
Formamide	−1.6	0.39	0.34	0.98
Methanol	−0.2	0.81	0.70	0.93
Acetone	1.3	2.18	1.88	0.77
Nitromethane	9.4	2.37	1.96	0.67
DCE	9.5	3.98	3.51	0.32
Pyridine	−13.6	2.12	1.43	0.43

The four worst‐performing solvents fared worse in every metric, reflecting both worse agreement for the majority of predictions and the presence of more numerous outliers. The results for these four solvents, along with reasons for the high error, are described later in this manuscript.

### 3.2 Evaluation of model on external test data

For each of the six solvents shown in Figure 3, we obtained additional experimental $pK_{a}$ data, filtering out any data that were used to fit the $\delta_{H}^{†}$ parameters.

### 3.3 Description of test data

Data were selected from seven sources:

- Zevatskii (2009):^51^ Various small acids and bases: 11 in water, six in methanol, seven in ethanol, and 11 in formamide.
- Cantu (2005):^52^ Four basic drug molecules and their $pK_{a}$ values in water, methanol, and acetonitrile.
- Sirén (2005):^53^ Five acidic neurotransmitter derivatives and 12 basic β‐blocker drugs with $pK_{a}$ values in water and methanol.
- Headley (1994):^54^ Eight simple carboxylic acids with values in water, methanol, and ethanol.
- Ludwig (1986):^55^ Benzoic acid derivatives and their $pK_{a}$ values: 27 in water, 26 in methanol, 27 in ethanol, 27 in DMF, and 25 in acetonitrile.
- Hughes (1986):^56^ Amino acids and two basic small molecules, totaling six values in water and 9 in DMSO. The remaining entries without aqueous data were supplemented by aqueous $pK_{a}$ values from the IUPAC Digitized $pK_{a}$ Dataset.^11^
- Ogston (1935):^57^ Three amino acids and one small molecule, in water and methanol, and one amino acid in methanol.

There were two cases in which different values were available from two sources. The maximum difference in $pK_{a}$ was 0.4 $pK_{a}$ units, so we used the average of their values in these two cases. We then further curated the data as was done for the parameterization set (see SI).

**FIGURE 4:**
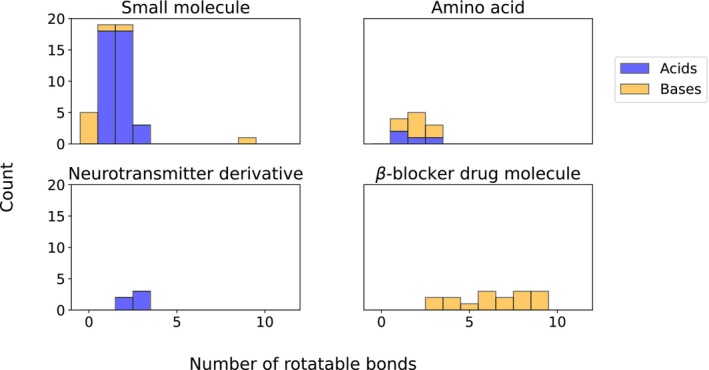
Distribution of the number of rotors in the test sets, demarcated by the class of molecule. The small molecule with the large number of rotors is tributylamine.

The test compounds include small acids and bases, much like the set of compounds used for parameterization, but also includes drug molecules with many rotatable bonds as well as amino acids, which we represent in their zwitterionic form in the QM calculations. Figure 4 shows the number of rotatable bonds for the molecules in the test set; all but one of the small molecules have fewer than four, whereas nearly all of the basic drug molecules have four or greater.

Using the same modeling methodology as described previously, and using the shift parameters obtained from the previous fitting (Table 2), we computed the $pK_{a}$ values and compared them to the literature data.

#### 3.3.1 Test results

**FIGURE 5:**
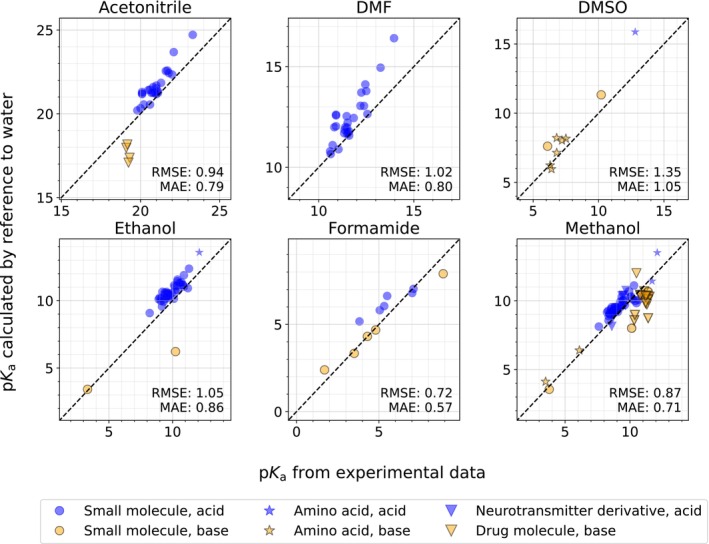
Test of $pK_{a}$ values calculated relative to their experimental data, for the six solvents depicted in Figure 3. The MAE and RMSE are shown in $pK_{a}$ units.

Figure 5 shows the parity between our predictions and the test data. The predicted $pK_{a}$ values show satisfactory agreement in most solvents, especially for small molecules. In fact, for solvents with only small molecules tested, we observed RMSEs of less than 1.1 $pK_{a}$ unit. The best performance was in formamide, with an RMSE of 0.72 $pK_{a}$ units. Also, in DMF, formamide, and DMSO, the $pK_{a}$ values come from only one source, which further minimizes the chance of systematic error from different acidity scales within the same solvent (such systematic error can be quite significant, on the order of several kcal mol^−1^, and is discussed later). Hence, the remaining uncertainty is due to the quantum chemical calculations as well as any inherent aleatoric uncertainty in the reference data.

We focus here on a few outliers. First, in acetonitrile and methanol, several basic drug molecules showed a significant underprediction compared to the acids, which were generally overpredicted. This follows a pattern seen in Figure 3, where the same behavior was generally seen for acids and bases, albeit to a lesser extent. Similarly, in ethanol, there was a large underprediction in the $pK_{a}$ of tribuytlamine, which has a large number of conformers (the rightmost bar in the small molecule section of Figure 4). Hence, the molecules with the largest number of rotors tended to have much larger errors using this method. Note that in our computational workflow, we limited the number of conformers in the COSMO‐RS calculation to 20 or fewer, as finding numerous thermodynamically significant conformers for very large molecules is a time‐expensive task. However, it is not clear whether conformer effects are primarily responsible for these errors, as experimental inconsistencies, COSMO‐RS limitations based on theory or parameterization, and incomplete sets of conformers are all implicated in systematic error. Future work should elucidate the effect of conformers on the efficacy of this method.

Despite the underprediction of $pK_{a}$ values for large molecules, we observed generally good agreement between our calculations and the reference data, including for zwitterionic amino acids and neurotransmitter derivative molecules. We thereby recommend using this method to determine acidities and basicities of small organic molecules and amino acids in acetonitrile, DMF, DMSO, ethanol, formamide, and methanol.

### 3.4 Sources of error

**FIGURE 6:**
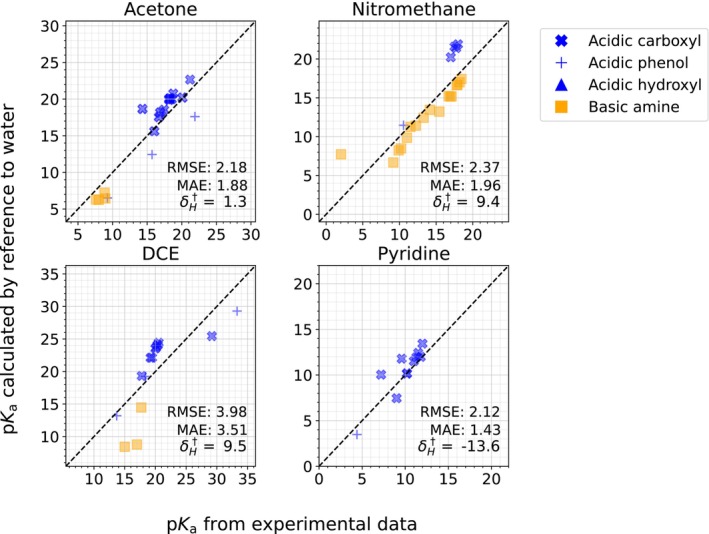
$pK_{a}$ values calculated relative to a reference $pK_{a}$, for the four solvents with MAEs greater than 1 $pK_{a}$ unit. The RMSE and MAE are shown in $pK_{a}$ units, and the corresponding $\delta_{H}$ in kcal mol^−1^. Data are colored by functional site of the ionization center.

As discussed previously, this method was also used to parameterize acetone, nitromethane, DCE, and pyridine, but the model agreement was observed to be worse. Figure 6 shows the poor quality of the model predictions during the parameterization step.

This occurs in some part due to inconsistencies in experimental data. $pK_{a}$ data in non‐aqueous solvents are prone to systematic errors; they can deviate due to inconsistent calibration methods, as $pK_{a}$ values in a solvent are typically measured with reference to each other and then anchored to an absolute scale. Another potential source of error comes from the solvation model, which previous work has shown can systematically differ in solvation free energies by several kcal mol^−1^ based on the ionization center.^34^ Although we curated the data to reduce the occurrence of data‐sourced errors, it is possible that several errors are still present. Furthermore, there are experimental considerations such as ion pairing, which occurs in low‐polarity solvents such as pyridine and DCE, and may further contribute to error.^33^ On the basis of our data curation, we labeled the experimental data in nitromethane and DCE as unreliable, but still include our predictions herein in the absence of more reliable values for demonstrative purposes. For a more rigorous discussion of the data curation, we refer readers to the SI.

#### 3.4.1 Experimental data for nitromethane

We observed that carboxylic acid $pK_{a}$ values were overpredicted compared to the amine and phenol values (Figure 6).

The carboxylic acids are benzoic acid derivatives from the same single source, anchored to the $pK_{a}$ value of 10.5 for picric acid. The $pK_{a}$ used in this work for benzoic acid is 17.73^56^ and for picric acid (the only phenol in this solvent) the $pK_{a}$ was 10.5.^59^ However, values reported elsewhere on the same acidity scale have included 13.2 for benzoic acid and 7.94 for picric acid.^60^, ^61^ The high deviations for carboxylic acids therefore cannot be immediately explained.

The amine values are anchored to a $pK_{a}$ of 17.2 for 1,3‐diphenylguanidine. This is possibly a different scale than the one for the acids. The Izutsu $pK_{a}$ compilation^16^ reports values of 13.2 for benzoic acid when anchored to this value of 17.2 for the amines; but the value for benzoic acid used in our work was 17.73, which would imply that the benzoic acids are systematically misaligned from amines by 4.5 $pK_{a}$ units. This is approximately the same amount by which acidic carboxylic acids were overpredicted in Figure 6. Therefore, we expect that anchoring the experimental data to the same scale in nitromethane would improve the performance of the COSMO‐RS method.

Even if the acidity scales were to be aligned, the nitromethane data still overall remain questionable, as the shift parameters estimated herein are vastly different from estimates obtained from comparisons of transfer energies from water to nitromethane and from water to acetonitrile (see SI). Hence, we assess the nitromethane $pK_{a}$ data in general to be highly unreliable and do not recommend its usage.

#### 3.4.2 Experimental data for DCE

In DCE, generally poor agreement was observed, with amines significantly underpredicted compared to carboxylic acids. The $pK_{a}$ values for the carboxylic acids and picric acid come from the same collection of papers^62^, ^63^, ^64^, ^65^, ^66^ (except for acetic acid at pH = 29.2, the outlier on the right of Figure 6), and are anchored to the $pK_{a}$ of hydriodic acid = 7.9 (corresponding to $pK_{a}$ of picric acid = 13.7). The $pK_{a}$ values for the three bases are also anchored to the same value.^67^ Therefore, most of the data in DCE is aligned. We could not determine the sources for the other two phenols and for acetic acid.

Note, further, that theoretical values for the dissociation of picric acid have been reported to be as high as 45.0, if the experiment is done with a completely pure and dry sample, which would strongly disagree with the scale followed by all other values herein.^33^

In summary, the data appear to be almost, if not fully, anchored to the same scale. Therefore, the issue of consistent anchor values cannot solely explain the poor model performance. DCE is known to be a solvent with considerable experimental challenges including ion pairing, and therefore, we consider this set of data to also be unreliable.

#### 3.4.3 Model error

An unfortunate limitation of this method is that experimental data and model error cannot be decoupled. Therefore, there also remains the possibility that error from the solvation model (e.g., limitations of modeling ions, insufficient sampling of conformers) is a significant contributor to error. Previous work^34^, ^68^ has shown that COSMO‐RS predictions of ionic solvation energies deviate from experimental predictions by an offset that is systematically too high for cations and too low for anions. A single parameter added to the model predictions (with a different sign depending on the ion's charge) accounted for most of that offset. However, it is not clear how much of the offset arises from uncertainty in anchoring the proton's solvation energy versus from model accuracy. Because the value of that optimal parameter changes significantly depending on the model type, we believe it possible that a portion of the systematic deviation is from model error. In Equations (1) and (2), the difference in anion solvation energies between two solvents is *added* while the difference for cations is subtracted; so, any such oppositely‐signed systematic model error would manifest as a systematic offset in $\delta_{H}^{†}$ as well. Previous work has also shown that there is systematic error based on the functional group at the ionization center; this may contribute to the poor prediction quality in some of the solvents that appear to be dependent on the type of acid.^34^

We emphasize here that our workflow lumps together model error with experimental error. This has two important consequences: (1) unambiguously discerning the cause of outliers is difficult, if not impossible, and (2) values of $\delta_{H}^{†}$ may not be understood as accurate, empirical determinations of $\Delta \Delta G_{\text{solv}}^{†}\left(H^{+}\right)$ but rather as estimates. Recovering a reliable value of the proton transfer energy based on $\delta_{H}^{†}$ implies that $\Delta \Delta G_{\text{solv}}\left({\mathrm{BH}}_{i}^{+}\right)$ and $\Delta \Delta G_{\text{solv}}\left(A_{i}^{-}\right)$ computed using COSMO‐RS have negligible systematic error, and all experimental $pK_{a}$ data are anchored to consistent anchor values, both internally (i.e., the same acidity scale for all compounds in a single solvent) and among solvents (i.e., the chosen anchor value for each solvent is based off of sensible extrathermodynamic assumptions, whether implicitly or explicitly). These assumptions are certainly not perfectly valid, so to probe the strength of them, we compare our computed values of $\delta_{H}^{†}$ to proton transfer energies derived using separate extrathermodynamic assumptions.

### 3.5 Comparison of $\delta_{H}^{†}$ to proton transfer energies in literature

We compared our computed values of $\delta_{H}^{†}$ to experimental values of $\Delta \Delta G_{\text{solv}}^{†}\left(H^{+}\right)$ from water. In the most ideal case, the values of $\delta_{H}^{†}$ should exactly equal the difference in solvation energies of the proton ($\Delta \Delta G_{\text{solv}}^{†}\left(H^{+}\right)$). However, this is complicated by the fact that any systematic error in COSMO‐RS corrections will also contribute to $\delta_{H}^{†}$, as will any systematic errors in $pK_{a}$ reference data. Furthermore, the choice of anchor value in non‐aqueous solvents is based on consistency with extrathermodynamic assumptions. Hence, any proton transfer energy that is recovered from this approach will agree most strongly with the value corresponding to the extrathermodynamic assumption used to anchor the scale. That said, comparing our computed values to proton transfer energies reported in the literature could at least provide a loose estimate for how much of the term $\delta_{H}^{†}$ originates from correcting for the proton solvation energies versus from systematic error.

Most efforts in the literature are concentrated around just a few solvents, limiting the scope of our comparison.^69^ Additionally, depending on the extrathermodynamic assumption invoked in fixing the energy scale, different values derived from experiments have been reported for each solvent.^40^ Values have been reported using the tetraphenylarsonium‐tetraphenylborate (TATB) assumption,^70^, ^71^ the cluster‐pair approximation (CPA),^27^, ^28^, ^72^, ^73^ and by comparing experimental and computed $pK_{a}$ values (Rossini and this work).^25^, ^26^, ^29^, ^30^ We refer interested readers to the corresponding references for more information about these approaches.

Table 3 shows that our $\delta_{H}^{†}$ parameters showed general agreement with experimental transfer energies of the proton in several solvents. There is no single method or solvent that agrees perfectly with our values; for instance, our values for acetonitrile and methanol agree well with the Rossini values, but disagree with the transfer energy reported in DMSO. The ordering of very positive values (acetonitrile) and very negative values (DMSO, DMF) are in agreement across all methods, while the intermediate values show considerably greater deviation.

**TABLE 3:** Comparison of $\delta_{H}^{†}$ to consensus proton transfer energies derived from experiment (kcal mol^−1^).

Solvent	$\delta_{H}^{†}$	$\Delta \Delta G_{\text{solv}}^{†}\left(H^{+}\right)$ (TATB)^35^	(CPA)^27^	(Rossini)^29^, ^30^
Acetonitrile	10.1	10.7	5.7	10.8
DMSO	−6.1	−4.6	−7.4	−0.5
Ethanol	−1.5	2.7	–	–
Methanol	−0.2	2.1	2.4	0.0
DMF	−6.9	−3.4	–	–
Formamide	−1.6	–	–	–

The Rossini estimates were derived using a similar approach to the one presented herein, but were computed by adding solvation energy corrections to quantum‐chemical calculations rather than to reference $pK_{a}$ values. Our estimates for $\delta_{H}^{†}$ appeared to match closely with Rossini's estimates except for in the case of DMSO, in which case our estimates match the TATB and CPA estimates far more closely. Values for ethanol and DMF could only be compared to energies obtained from the TATB assumption. These estimates were also comparatively worse than those in other solvents, with differences of 4.2 kcal mol^−1^ for ethanol and 3.5 kcal mol^−1^ for DMF compared to estimates using the TATB assumption. Though there are substantial deviations among the estimates for proton transfer energies, our computed values of $\delta_{H}^{†}$ roughly agree with the proton transfer energies estimated in the literature. These results suggest that the $\delta_{H}^{†}$ regression parameter generally captures the proton transfer energy, and can be used to approximate such energies in solvents where other estimates are unavailable.

Because we observed a good quality fit for $\delta_{H}^{†}$ and good test performance in formamide, we assign an estimate of the proton transfer energy from water to formamide as −1.6 kcal mol^−1^.

To our knowledge, there is no consensus value for the proton's transfer energy in formamide for any given extrathermodynamic assumption. Values reported in the IUPAC compilation of transfer free energies from water include 1.0, −3.3, −4.1, and −1.7 kcal mol^−1^, which on average satisfatorically agree with the value we report herein; however, these values were considered “unsatisfactory”, and do not reflect a consensus value.^74^ The transfer energy reported above should be considered as an estimate, not a definitive value, and reflect the underlying assumptions used to construct the pH scale in the reference data. We further advise against using the $\delta_{H}^{†}$ obtained in this work to estimate proton transfer energies for acetone, nitromethane, pyridine, and DCE, as in these solvents, our linear regression was unable to determine the fitting parameters satisfactorily.

## 4 CONCLUSION

We have discussed how experimental acid dissociation constants in one solvent can be combined with COSMO‐RS solvation energy calculations to compute the $pK_{a}$ in a different solvent. This technique requires an estimate of the proton's solvation energy in each solvent, and in this work we propose a way to estimate the needed proton transfer energies via regression. Our computed proton transfer energies are in rough agreement with other estimates in the literature. During parameterization, we observed residuals within 1 $pK_{a}$ unit of MAE for 6 solvents using this method. Errors are larger for 4 other solvents—in those cases, a large portion of error is likely due to poor data quality. Further, we tested this method to predict the acidities and basicities of an external set of compounds in acetonitrile, DMF, DMSO, ethanol, formamide, and methanol. We observed generally good agreement, with MAE <1.1 $pK_{a}$ unit in all of those six solvents. The $pK_{a}$ values of small molecules were predicted particularly accurately, but values for bases with large numbers of rotatable bonds were consistently underpredicted by several $pK_{a}$ units. One potential reason is that the computational workflow used herein restricted the number of conformers in the COSMO‐RS method to 20 or fewer; however, it is difficult to disentangle conformer effects from uncertainty due to experimental error and limitations of the implicit solvation model method. In addition, it is possible that performing the conformer search in only the aqueous phase instead of other solvents may affect prediction accuracy. The potential for conformer search in different solvents to affect computed solvation free energies has been observed in previous work for neutral solutes,^75^ and is not yet well‐understood for ionic solutes. Future work should investigate the extent of these effects for $pK_{a}$ prediction, particularly in the four solvents that were identified to have poor performance.

We also used this method to estimate the transfer free energy of the proton from water to formamide. We emphasize that the proton transfer free energy is implicitly or explicitly assumed when establishing the pH scale in non‐aqueous solvents, and thus depends entirely on the anchor values used in anchoring the acidity scales. One disadvantage to this method is that at least one reference $pK_{a}$ is required for each desired solvent system. Furthermore, potentially time‐expensive QM calculations are required to generate the conformers used in the COSMO‐RS calculation, especially for very large molecules. However, we are optimistic that ongoing advances in data availability and processing power will reduce the impact of these obstacles. Because the method described herein relies only on calculations of solvation free energies, any solvation model that can consider ionic solutes can be used. Future efforts should investigate the performance of this method when employing other solvation models, such as the implicit Minnesota SMx models^76^, ^77^, ^78^ or cluster‐continuum solvation methods.^79^

A key challenge for using this approach is the lack of high‐quality reference data in many solvents. We hope that large datasets of dissociation constants in diverse solvent systems with unified acidity scales will become available, enabling the computation of useful thermochemical properties in more solvents. One other barrier is the lack of consensus around the use of extrathermodynamic assumptions in accurately determining proton solvation energies. We hope that future research can glean insight into the merits of the different extrathermodynamic assumptions and their effects in anchoring energy scales.

## Supporting information


**Data S1:** supporting Information.

## Data Availability

The data that support the findings of this study are openly available in Supporting materials for: pKa Prediction in Non‐Aqueous Solv at https://zenodo.org/doi/10.5281/zenodo.11153563.
