# Supplementary material for: pK a prediction in non‐aqueous solvents
Source: J Comput Chem. 2024 Dec 11;46(1):e27517. doi: 10.1002/jcc.27517 (PMC11633825; doi:10.1002/jcc.27517)
Supplement: Supplementary file 1 — Data S1: supporting Information. [file JCC-46-0-s001.pdf]

Supporting Information for: pK_a Prediction in Non-Aqueous Solvents

Jonathan W. Zheng,[†] Emad Al Ibrahim,[†] Ivari Kaljurand,[‡] Ivo Leito,[‡] and William H. Green*,[†]

†*Department of Chemical Engineering, Massachusetts Institute of Technology, Cambridge, MA, 02139, U.S.A.*

‡*Institute of Chemistry, University of Tartu, 50411 Tartu, Estonia*

E-mail: whgreen@mit.edu

Phone: +1 617 (253)-4580

Supporting Information: Data Curation

General comments on curation of the pK_a data

The curation of the pK_a data in the solvents included in this work involved the following:

1. The pK_a measurement methods as presented in the original papers were examined. Particular attention was paid to how the pH measurement (involved in most pK_a measurements) was calibrated (i.e., which calibration compounds were used and with which pK_a values).

2. Attention was paid to whether the pK_a values change in logical manner when comparing pK_a values of related compounds in the same solvent or pK_a values of the same compound in different solvents:

(a) Within the same solvent, it was examined whether the differences between pK_a values of compounds correspond logically to the differences in their structures. An example: except possibly in very special cases, a chloro-substituted compound is a stronger acid than the similar methyl-substituted compound but weaker than the similar nitro-substituted compound.

(b) In the case of pK_a values of the same compound in different solvents it was examined whether the pK_a differences are logical considering the typical solvation Gibbs energies of the participating species in the different solvents. Most of the time the experiment-derived transfer Gibbs energies[1,2] from water, e.g. $\Delta_{tr}G^o(\text{Species}, \text{H}_2\text{O} \rightarrow \text{S})$ were used because their differences correspond to differences in solvation Gibbs energies. In some cases also the Minnesota Solvation Database[3] was used. As an example, just because the $\Delta_{tr}G^o(\text{H}^+, \text{MeCN} \rightarrow \text{DMSO})$ is -15.8 kcal mol^{-1} (corresponding to 11 orders of magnitude) and the transfer Gibbs energies of anions and neutrals are not very different, the pK_a of uncharged acids in MeCN are (almost) always 9-12 pK_a units higher than in DMSO.

3. In the case of some solvents useful collections of pK_a values or high-quality reference works are available: in water the collections by Kortüm et al[4] and Perrin;[5] in DMSO, the large body of work by the Bordwell group;[6] in acetonitrile, the self-consistent pK_a scales of acids[7] and bases;[8] in DMF a paper by the Kolthoff group;[9] in the case of acetone the paper by Foltin et al.[10] Some of them contain quality assessments[4,5] of pK_a values, some present carefully constructed self-consistent pK_a scales.[6–8] The pK_a values of other authors were examined, comparing them to reliable pK_a values from these works an taking into account structural differences. As a result of curation some pK_a values were left out of the training and test data sets and some values were corrected. More comments are below, with specific solvents.

Protic solvents: Water, methanol, ethanol and formamide

The four protic solvents (S) – water, methanol (MeOH), ethanol (EtOH), and formamide (FA) - have relatively high relative permittivity and good ability to solvate both cations and anions. All four are also reasonably strong hydrogen bond donors. As a result, acid dissociation according to the Brønsted scheme is favored in all four solvents. Because of the properties these solvents acids dissociate readily in them and their ability to support acid dissociation is not very sensitive to impurities (of which water is the most important in the case of MeOH, EtOH and FA). Thus, pK_a measurement in these four solvents is not generally challenging.

The transfer Gibbs energies of ions $\Delta_{tr}G^o(\text{ion}, H_2O \rightarrow S)$ from water into the three non-aqueous solvents are mostly between +2 and +5 kcal mol^{-1}.[1] In the case of MeOH and EtOH the $\Delta_{tr}G^o(H^+, H_2O \rightarrow S)$ is +2.5 and +2.7 kcal mol^{-1}, respectively. The $\Delta_{tr}G^o(H^+, H_2O \rightarrow FA)$ value is not known conclusivley but taking into account its other basicity parameters it is expected to be around zero or slightly negative. The $\Delta_{tr}G^o(\text{neutral}, H_2O \rightarrow S)$ are in the case of most polar molecules close to zero and in the case of low polarity molecules can be slightly negative. These data suggest that the pK_a values of uncharged acids in MeOH, EtOH and FA are higher than in water, pK_a in EtOH being the highest and pK_a in FA being the lowest. This is indeed what is observed in the majority of cases. The pK_a values in MeOH are mostly 4 to 6 pK_a units (depending on compound class) higher than in water and the pK_a values in EtOH are around 0.5 to 1.5 pK_a units higher than in MeOH. The pK_a values in FA in are on an average 2 pK_a units higher than the values in water.

In the case of cationic acids BH^+ the dissociation equilibrium involves transformation of one ionic species (BH^+) to another one (SH^+). Thus, to a significant part the $\Delta_{tr}G^o$ values cancel. Thus, the pK_a values of cationic acids in all four solvents should be similar. This is indeed what is observed: the pK_a values of cationic acids in MeOH, EtOH and FA are typically within ±2 pK_a units from the aqueous values. The pK_a values of BH+ in FA are typically lower than those in water and the pK_a values of BH^+ in MeOH and EtOH are

typically higher.

In the case of pK_a values of well-known compounds in water, Kortüm et al[4] and Perrin[5] collections were consulted if several pK_a values were available in the literature.

DMSO, MeCN, DMF

All three solvents have relatively high permittivity. DMSO and DMF are also rather basic, their $\Delta_{tr}G^o(H^+, H_2O \rightarrow S)$ being -4.61 and -3.32 kcal mol^{-1}, respectively as reported by Marcus. MeCN is significantly less basic, with $\Delta_{tr}G^o(H^+, H_2O \rightarrow \text{MeCN}) = +11.1$ kcal mol^{-1}.[1] The $\Delta_{tr}G^o(X^-, H_2O \rightarrow S)$ vary widely in all three solvents depending on the anion but are similar for the same anion between the solvents.[1] Given these properties of the solvents, the pK_a values of uncharged acids in DMSO and DMF are similar, with pK_a values in DMF somewhat higher. The pK_a values of uncharged acids in MeCN are, depending on anion solvation effects by 9-12 pK_a units higher than in DMSO. In the case of cationic acids the pK_a values in DMSO and DMF are very similar and the pK_a values in MeCN are typically by 6-10 pK_a units higher. Differently from the above mentioned protic solvents, the pK_a measurements in these solvents, especially acetonitrile, are quite sensitive to impurities, especially to water. If insufficiently dry solvent is used then biased (lower) pK_a values are obtained. The effect of traces of water can be to a large extent eliminated if relative measurement methods[7,8] are used.

In DMSO, the "primary reference" of evaluation was the large body of pK_a values by the Bordwell group,[6] which logically follow the structural effects and can be considered highly reliable. Similarly, in the case of acetonitrile the self-consistent pK_a scales of uncharged[7] and cationic[8] acids are considered as the most reliable and compounds not present there are evaluated against these scales considering the effects of molecular structure on pK_a. In DMF there are certain high-quality works[9] that provide reference values used by others.

Acetone

The transfer Gibbs energy value $\Delta_{tr}G^o(H^+, H_2O \rightarrow AC)$ of the proton and its Gibbs energy of solvation in acetone (AC), are not known but on the basis of basicity (electron donor) parameters, such as the Kamlet-Taft β[11] it can be estimated that acetone is somewhat more basic than acetonitrile. At the same time its relative permittivity and anion-solvating ability[1] are lower. The $\Delta_{tr}G^o(X^-, H_2O \rightarrow S)$ are mostly 1 to 2 kcal mol^{-1} more positive (for the halide ions), compared to acetonitrile. Consequently, pK_a values of uncharged acids in acetone are expected to be similar or somewhat lower than those in acetonitrile. This is indeed observed in most cases.

As far as comparison is possible, the transfer energies of cations $\Delta_{tr}G^o(\text{cation}^+, H_2O \rightarrow AC)$ and $\Delta_{tr}G^o(\text{cation}^+, H_2O \rightarrow \text{MeCN})$ are similar. This, together with the somewhat higher basicity implies that the pK_a values of cationic acids in AC should be slightly lower than in MeCN. This, however, not what is observed in most cases in the original data. Most of the pK_a data for cationic acids in AC are by 4 to 7 pK_a units lower than in MeCN. Almost all the cationic acid pK_a data in AC data are referred to the papers of Zevatskii et al[12] and Carabias-Martínez et al.[13] However, neither of these authors measured the pK_a values themselves, but obtained them from literature. Most (but not all) of the data come from different Russian authors. Like the previous group of aprotic solvents, the pK_a measurements in acetone are also strongly affected by water in the solvent, leading to downward bias in pK_a values.

At the same time, it the paper of Foltin et al[10] where the pK_a of picric acid – which serves as the primary reference for most of the pK_a values in acetone – is measured with high care, also the pK_{aH} values of 2- and 4-nitroaniline have been measured as 4.82 and 5.90, respectively. These values are very close to the respective values in acetonitrile 4.80 and 6.22, which is what is expected on the basis of transfer Gibbs energies. There are also works by Aufauvre et al where the pK_{aH} values of e.g. aniline, 2-methylaniline and N-methylaniline are reported as 8.84, 8.95 and 7.62, all higher than the respective values in the references[12,13]

(5.92, 5.94, 5.6) and are all much closer to the values in MeCN (10.64, 10.49, 10.97). Thus, all the low pK_a values of cationic acid in AC were regarded as questionable and left out of the data analysis.

Pyridine (Py)

The $\Delta_{tr}G^o(H^+, H_2O \rightarrow Py)$ is by 2.2 kcal mol^{-1} more negative than water to DMSO. At the same time, $\Delta_{tr}G^o(A^-, H_2O \rightarrow Py)$ is about 1.3 to 2.3 kcal mol^{-1} less positive than to DMSO. Assuming the transfer free energies of neutrals are small compared with those of ions, and that at low concentrations ion pairs in pyridine dissociate, the pK_a values in Py and DMSO are expected to be not too different. This is what is observed when looking at the pK_a values of uncharged acids.

The pK_a values of the two charged acids (butylammonium and triethylammonium) are by around 5 orders of magnitude lower than the respective DMSO values, which looks unlikely. The values are referred to Carabias-Martínez et al.[13] who, however, did not measure them. Both values were regarded as questionable.

Nitromethane (NM)

The $\Delta_{tr}G^o(H^+, H_2O \rightarrow NM)$ is by around 11.7 kcal mol^{-1} more positive than water to MeCN.[1] This is supported by a COSMO-RS calculation yielding 10.3 kcal mol^{-1}. Thus, NM is significantly less basic as a solvent than MeCN. At the same time, $\Delta_{tr}G^o(A^-, H_2O \rightarrow NM)$ are similar to MeCN.[1] Assuming the transfer free energies of neutrals are small compared with those of ions, the pK_a values of uncharged acids in NM should be on an average 7-10 pK_a units higher than in MeCN. The $\Delta_{tr}G^o(\text{cation}^+, H_2O \rightarrow NM)$ values seem to be on an average somewhat more positive than in MeCN, meaning that the differences between pK_a values of cationic acids between these two solvents could be somewhat smaller but the pK_a values in NM should still be higher than in MeCN.

The situation observed in the table is very different. The NM pK_a values of all uncharged

acids in the table are either equal or lower than those in MeCN. This is difficult to explain by anything else than experimental problems. On the one hand, like the previous group of aprotic solvents, the pK_a measurements in NM are also strongly affected by water in the solvent, leading to downward bias in pK_a values. On the other hand, NM is not a stable solvent; from prior experience, only fresh solvent is usable. Given the low basicity of NM, any impurity that is even slightly basic can make the pK_a values a lot lower than they should be in the pure solvent.

Thus, all the pK_a values in NM have been marked as questionable.

1,2-dichloroethane (DCE)

Given the extremely low basicity of DCE, it is not expected that the true Brønsted process of acid dissociation, involving protonation of the solvent, is experimentally observable. Almost any impurity in DCE is a much stronger base than DCE. The most important example is water. As an example, a realistic pK_a for anhydrous DCE (i.e. for the situation where DCE itself is protonated) is 45[14] which is most likely not measurable experimentally, because the solvent always contains at least some amount of water.

Given this, all the presented values are off by orders of magnitude, if they are to be interpreted as pK_a values referring to the Brønsted process. They are most likely relative values or massively (by many orders of magnitude) in error. Curiously, some of the DCE pK_a values in the table are even lower than the respective pK_a values in MeCN.

All the DCE values have been rejected from data analysis.

Supporting Information: Reference Ensembling

This section investigates the potential of using multiple references for the prediction of pK_a. As discussed in the manuscript, pK_a values from different sources might have systematic errors which make their use as reference values (p${K_a}^{ref}$) unreliable. One way to mitigate the

effect of potentially erroneous data is the use of an ensemble of reference values. Since all the $\delta^{\dagger}$ values reported in Table 2 in the main text are calculated with the same reference (water), we can infer the $\Delta\Delta G_{\text{solv}}^{\text{ref}}(\text{H}^+)$ term by simply using the difference of $\delta^{\dagger}$ values between the solvent and the new reference:

$$\delta^{\dagger}_{\text{solvent}} - \delta^{\dagger}_{\text{ref}} = \Delta G_{\text{solv}}^{\text{solvent}}(\text{H}^+) - \Delta G_{\text{solv}}^{\dagger}(\text{H}^+) - \Delta G_{\text{solv}}^{\text{ref}}(\text{H}^+) + \Delta G_{\text{solv}}^{\dagger}(\text{H}^+) = \Delta\Delta G_{\text{solv}}^{\text{ref}}(\text{H}^+) \tag{S1}$$

Therefore, the pK_{a} of a solute in a solvent can be calculated by applying solvent corrections to pK_{a} values of the solute in all available reference solvents and taking the average. For acids, this relationship is:

$$\text{p}K_{\text{a}}{}^{\text{solvent}} = \frac{1}{\text{N}_{\text{ref}}}\sum_{n}^{\text{N}_{\text{ref}}}\left(\text{p}K_{\text{a}}{}^{\text{ref}_{\text{n}}} + \frac{1}{2.303\text{RT}}\Big(\Delta\Delta G_{\text{solv}}^{n}(\text{H}^+) + \Delta\Delta G_{\text{solv}}^{n}(\text{A}^-) - \Delta\Delta G_{\text{solv}}^{n}(\text{AH})\Big)\right) \tag{S2}$$

and for bases,

$$\text{p}K_{\text{a}}{}^{\text{solvent}} = \frac{1}{\text{N}_{\text{ref}}}\sum_{n}^{\text{N}_{\text{ref}}}\left(\text{p}K_{\text{a}}{}^{\text{ref}_{\text{n}}} + \frac{1}{2.303\text{RT}}\Big(\Delta\Delta G_{\text{solv}}^{n}(\text{H}^+) - \Delta\Delta G_{\text{solv}}^{n}(\text{BH}^+) + \Delta\Delta G_{\text{solv}}^{n}(\text{B})\Big)\right) \tag{S3}$$

where N_{ref} refers to the total number of available reference solvents, $\Delta\Delta G_{\text{solv}}^{n}(Z) \equiv \Delta G_{\text{solv}}^{\text{solvent}}(\text{Z}) - \Delta G_{\text{solv}}^{\text{ref}_{\text{n}}}(\text{Z})$, and p$K_{\text{a}}{}^{\text{ref}_{\text{n}}}$ refers to the dissociation corresponding to p$K_{\text{a}}{}^{\text{solvent}}$ in the nth reference.

Table S1 shows a comparison between the use of water as a reference (labeled as H_2O) as opposed to an ensemble of references (labeled as mean). It should be noted that although aqueous pK_{a} data is available for the whole dataset, many solutes have missing pK_{a} values in other references. The results shown in Table S1 will thus depend on the available data for a given solute in water, acetonitrile, DMF, DMSO, ethanol, and methanol. The benefits of ensembling can be non-trivial as shown by the noticeable reductions in the mean absolute

errors for most solvents as compared to only using water as a reference. Note that the data used for the fitting is identical to that shown in Figure 3 in the main text and the data used for prediction is shown in Figure 5 in the main text.

Table S1: Comparison between using a single reference in water and reference ensembling. The MAE is shown in kcal mol^{-1}.

**Solvent**	**MAE fitting**			**MAE prediction**		
	**H_2O**	**mean**	**mean$_s$**	**H_2O**	**mean**	**mean$_s$**
**Acetonitrile**	0.72	**0.58**	**0.58**	0.79	**0.32**	**0.32**
**DMF**	0.90	**0.63**	**0.63**	0.80	**0.49**	0.68
**DMSO**	0.95	0.91	**0.86**	**1.05**	**1.05**	**1.05**
**Ethanol**	0.87	0.71	**0.67**	0.86	0.48	**0.42**
**Formamide**	0.34	0.28	**0.27**	0.57	0.50	**0.46**
**Methanol**	0.70	0.66	**0.65**	0.71	**0.49**	0.50

The optimization procedure described earlier relies on aqueous data fits as shown in equation 4 in the main text. This does not make use of all the available data in other reference solvents. Moreover, the terms $\delta^{\dagger}_{\text{solvent}}$ and $\delta^{\dagger}_{\text{ref}}$ in equation S1 both have associated errors that might propagate to the $\Delta\Delta G^{\text{ref}}_{\text{solv}}(\text{H}^+)$ term. Alternatively, a simultaneous fit can be made in all available reference solvents where the first term ($\Delta G^{\text{solvent}}_{\text{solv}}(\text{H}^+)$) is estimated based on data from all references while each ($\Delta G^{\text{ref}}_{\text{solv}}(\text{H}^+)$) is estimated using data for its corresponding nth reference. The optimization problem will thus generalize to N_{ref} number of solvents as follows:

$$f_{n,i}(\boldsymbol{\delta}) = \begin{cases} \text{pK}^{\text{ref}_\text{n}}_{\text{a}_i} - \text{pK}^{\text{solvent}}_{\text{a}_i} + \frac{1}{2.303\text{RT}}\Big(\delta_0 - \delta_n - \Delta\Delta G^{n}_{\text{solv}}(\text{BH}^+_i) + \Delta\Delta G^{n}_{\text{solv}}(\text{B}_i)\Big) & \text{i} = \text{base} \\ \text{pK}^{\text{ref}_\text{n}}_{\text{a}_i} - \text{pK}^{\text{solvent}}_{\text{a}_i} + \frac{1}{2.303\text{RT}}\Big(\delta_0 - \delta_n + \Delta\Delta G^{n}_{\text{solv}}(\text{A}^-_i) - \Delta\Delta G^{n}_{\text{solv}}(\text{AH}_i)\Big) & \text{i} = \text{acid} \end{cases} \tag{S4}$$

$$\boldsymbol{\delta_H} = \text{argmin}_{\boldsymbol{\delta}}\left(\sqrt{\sum_{\text{n,i}}^{\text{N}_{\text{ref}},\text{N}} f_{n,i}(\boldsymbol{\delta})^2}\right) \tag{S5}$$

where i refers to the neutral form of the acid or base under consideration, N is the number of datapoints per solvent, n refers to a reference solvent in a list of N_{ref} solvents, $\boldsymbol{\delta}$ is a vector proxy estimate for the proton transfer energy of length $\text{N}_{\text{ref}} + 1$, $\delta_0 \equiv \Delta G^{\text{solvent}}_{\text{solv}}(\text{H}^+)$,

$\delta_n \equiv \Delta G^{\mathrm{ref}}_{\mathrm{solv}}(\mathrm{H}^+)$, and $f_{n,i}(\boldsymbol{\delta})$ is the loss function that corresponds to the optimal $\boldsymbol{\delta_H}$ when minimized in all references. In equation S4, the pK_{a} terms are experimental values, whereas the $\Delta\Delta G^{\mathrm{ref_n}}_{\mathrm{solv}}$ terms are computed using COSMO-RS in their corresponding solvents.

The new simultaneous optimization procedure is done for each solvent in water, acetonitrile, DMF, DMSO, ethanol, and methanol. Ensemble results from the new fitting (labeled as mean$_{\mathrm{s}}$) are compared to single and ensemble aqueous data fits in Table S1, showing small but somewhat consistent reductions of error as compared to ensembles using $\delta^{\dagger}$ values from equation S1. Note that the prediction data for DMSO had water as the only reference so its value is identical to mean and mean$_{\mathrm{s}}$. Considering these results, ensembling multiple references can reduce the residuals during fitting as compared to only using an aqueous reference, as the method was shown to decrease the error in all examined cases. As more data becomes available in non-aqueous solvents, the number of references in the ensemble is expected to grow and thus yield more pronounced effects.

References

(1) Marcus, Y.; Kamlet, M.; Taft, R. Linear solvation energy relationships: standard molar Gibbs free energies and enthalpies of transfer of ions from water into nonaqueous solvents. *The Journal of Physical Chemistry* **1988**, *92*, 3613–3622.

(2) Kalidas, C.; Hefter, G.; Marcus, Y. Gibbs energies of transfer of cations from water to mixed aqueous organic solvents. *Chemical reviews* **2000**, *100*, 819–852.

(3) Marenich, A. V.; Kelly, C. P.; Thompson, J. D.; Hawkins, G. D.; Chambers, C. C.; Giesen, D. J.; Winget, P.; Cramer, C. J.; Truhlar, D. G. Minnesota solvation database (MNSOL) version 2012. **2020**,

(4) Kortüm, G. *Dissociation constants of organic acids in aqueous solution*; Butterworth, 1961; Vol. 1.

(5) Perrin, D. D. *Dissociation Constants of Organic Bases in Aqueous Solutions*; Franklin Book Company, 1965; Vol. 1.

(6) Bordwell, F. G. Equilibrium acidities in dimethyl sulfoxide solution. *Accounts of Chemical Research* **1988**, *21*, 456–463.

(7) Kütt, A.; Tshepelevitsh, S.; Saame, J.; Lõkov, M.; Kaljurand, I.; Selberg, S.; Leito, I. Strengths of acids in acetonitrile. *European Journal of Organic Chemistry* **2021**, *2021*, 1407–1419.

(8) Tshepelevitsh, S.; Kütt, A.; Lõkov, M.; Kaljurand, I.; Saame, J.; Heering, A.; Plieger, P. G.; Vianello, R.; Leito, I. On the basicity of organic bases in different media. *European journal of organic chemistry* **2019**, *2019*, 6735–6748.

(9) Kolthoff, I. M.; Chantooni, M. K.; Smagowski, H. Acid-base strength in N, N-dimethylformamide. *Analytical Chemistry* **1970**, *42*, 1622–1628.

(10) Foltin, M.; Majer, P. Determination of dissociation constant of picric acid and calibration of potentiometric cell in acetone. *Collection of Czechoslovak Chemical Communications* **1978**, *43*, 95–102.

(11) Abboud, J.-L.; Notari, R. Critical compilation of scales of solvent parameters. Part I. Pure, non-hydrogen bond donor solvents. *Pure and Applied Chemistry* **1999**, *71*, 645–718.

(12) Zevatskii, Y. E.; Samoilov, D. Empirical method for consideration of solvent effect on the dissociation constants of carboxylic acids. *Russian journal of organic chemistry* **2008**, *44*, 52–61.

(13) Carabias-Martinez, R.; Rodriguez-Gonzalo, E.; Dominguez-Alvarez, J.; Miranda-Cruz, E. Development of a chemometric correlation technique to estimate acid–base

descriptors for cationic acids in non-aqueous media. *Analytica chimica acta* **2007**, *584*, 410–418.

(14) Paenurk, E.; Kaupmees, K.; Himmel, D.; Kütt, A.; Kaljurand, I.; Koppel, I. A.; Krossing, I.; Leito, I. A unified view to Brønsted acidity scales: do we need solvated protons? *Chemical Science* **2017**, *8*, 6964–6973.
